# Both Intrinsically Disordered Regions and Structural Domains Evolve Rapidly in Immune-Related Mammalian Proteins

**DOI:** 10.3390/ijms19123860

**Published:** 2018-12-04

**Authors:** Keiichi Homma, Hiroto Anbo, Tamotsu Noguchi, Satoshi Fukuchi

**Affiliations:** 1 Department of Life Science and Informatics, Maebashi Institute of Technology, 460-1 Kamisadori-machi, Maebashi-shi 371-0816, Japan; koume8@icloud.com (H.A.); sfukuchi@maebashi-it.ac.jp (S.F.); 2 Pharmaceutical Education Research Center, Meiji Pharmaceutical University, 2-522-1 Noshio, Kiyose-shi, Tokyo 204-8588, Japan; noguchit@my-pharm.ac.jp

**Keywords:** secretion, immune, extracellular, protein-protein interaction, intrinsically disordered region, structural domain, evolution

## Abstract

Eukaryotic proteins consist of structural domains (SDs) and intrinsically disordered regions (IDRs), i.e., regions that by themselves do not assume unique three-dimensional structures. IDRs are generally subject to less constraint and evolve more rapidly than SDs. Proteins with a lower number of protein-to-protein interactions (PPIs) are also less constrained and tend to evolve fast. Extracellular proteins of mammals, especially immune-related extracellular proteins, on average have relatively high evolution rates. This article aims to examine if a high evolution rate in IDRs or that in SDs accounts for the rapid evolution of extracellular proteins. To this end, we classified eukaryotic proteins based on their cellular localizations and analyzed them. Moreover, we divided proteins into SDs and IDRs and calculated the respective evolution rate. Fractional IDR content is positively correlated with evolution rate. For their fractional IDR content, immune-related extracellular proteins show an aberrantly high evolution rate. IDRs evolve more rapidly than SDs in most subcellular localizations. In extracellular proteins, however, the difference is diminished. For immune-related proteins in mammals in particular, the evolution rates in SDs come close to those in IDRs. Thus high evolution rates in both IDRs and SDs account for the rapid evolution of immune-related proteins.

## 1. Introduction

Mature eukaryotic proteins consist not only of structural domains (SDs), but also of intrinsically disordered regions (IDRs), i.e., regions that by themselves do not fold into unique three-dimensional structures [1]. Although some IDRs interact with proteins or other macromolecules, they are generally under less constraint than SDs and thus have higher evolution rates [2]. A positive correlation between fractional IDR contents of proteins and evolution rates is thus expected.

Proteins with more protein-to-protein interactions (PPIs) tend to be more evolutionarily constrained and have lower evolution rates [3,4]. Highly expressed proteins are also more constrained and evolve slowly [4,5,6]. These two factors partially account for the evolution rate of proteins.

Eukaryotic proteins have specific subcellular localizations in general, with different average fractional IDR contents in different cellular localizations [7]. For instance, IDR contents are generally high in nuclear proteins [7,8], while they tend to be low in mitochondrial proteins [9,10]. It is plausible that different fractional IDR contents in different subcellular localizations result in varied evolution rates.

Interestingly, extracellular proteins (synonymously called secreted proteins) in mammalian species were often found to evolve faster than intracellular proteins [11,12]. This finding is partly explainable by rapid evolution of immune-related extracellular proteins as many of the coding genes are subject to positive selection [13,14]. That is, the evolution rate, ω, defined by the nonsynonymous to synonymous substitution rate ratio, exceeds unity at sites under positive selection and the existence of many such sites result in high evolution rates of many immune-related genes. For instance, antimicrobial peptides, α- and β-defensins and cathelicidins, are reportedly subject to positive selection and evolve rapidly in mammals [15,16,17]. We consider it worthwhile to carry out research on evolutionary characteristics of immune-related secreted proteins, as they are involved in host defences [18], pathogen–host interactions [19,20], production of antibodies [21], colony-stimulating factors [22], haematopoiesis [23], and triggering proteolytic cascades [24,25], as well as enzyme replacement therapies [26]. The generally high evolution of immune-related proteins evinces their importance in evolution of mammalian species [27]. Further research may reveal how immune-related proteins function and may lead to pharmaceutical applications.

However, the difference in evolution rate with intracellular proteins remained significant even if analyses were limited to non-immune-related extracellular proteins. The generally low expression levels in secreted proteins partially explain the rapid evolution. Whether the substitution frequency in IDRs or SDs or both contributes to the increased evolution rate of extracellular proteins, however, has not been explored.

We examined the correlation of fractional IDR content and evolution rate and found it positive. We then analyzed the evolution rates of SDs and IDRs of proteins in different localizations. In most localizations, IDRs were found to evolve faster than SDs, as expected. Immune-related secreted proteins in mammals, however, exhibited extremely high evolution rates in SDs that approach those in IDRs. This surprising finding indicates that positive selection that is said to function on a number of immune-related genes operates strongly both on IDRs and SDs of the coded proteins.

## 2. Results and Discussion

### 2.1. Classification of Eukaryotic Proteins by Subcellular Localizations

For accurate analyses of evolution rates in different subcellular localizations, reliable localization annotations of most proteins are necessary. At present, only four species satisfy this criterion in UniProt: *Homo sapiens*, *Mus musculus* (mouse), *Arabidopsis thaliana* (thale cress), and *Saccharomyces cerevisiae* (budding yeast). We thus selected the human, mouse, thale cress, and budding yeast proteins with orthologs and classified the selected proteins by subcellular localization (Table 1). Proteins that are localized to both the nucleus and the cytosol were specifically grouped (abbreviated as NC), as the group reportedly contains many proteins with multiple PPIs [28]. We combined proteins residing in the endoplasmic reticulum and the Golgi apparatus (termed EG), since many proteins cycle between the two organells. Secreted proteins were divided into immune-related (SI) and non-immune-related (SN), because immune-related proteins generally evolve rapidly [11]. Thale cress had a limited number of immune-related proteins, while unsurprisingly budding yeast had none. Multiply localized proteins except for the aforementioned NC proteins were classified as one group (ML). Note that many proteins with orthologs were not classifiable due to the unavailability of pertinent information.

### 2.2. Evolution Rates and Other Properties of Proteins in Different Subcellular Localizations

**Figure 1:**
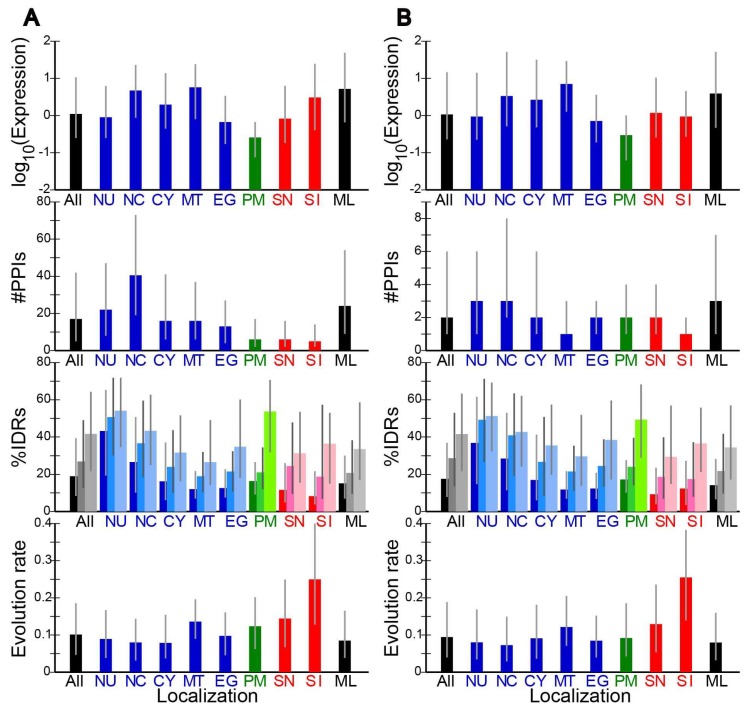
Medians and ranges of four quantities in different localizations in two mammals. (**A**) *H. sapiens*; (**B**) *M. musculus*; Rectangles in each panel from the bottom to the top represent the medians in evolution rate, fractional IDR content by DISOPRED (left), DICHOT (middle) and POODLE (right), the number of PPIs, and expression level. Grey vertical bars represent interquartile ranges, with their bottom and top corresponding to the 25th to the 75th percentile, respectively. The abbreviations for localizations are as in Table 1.

**Figure 2:**
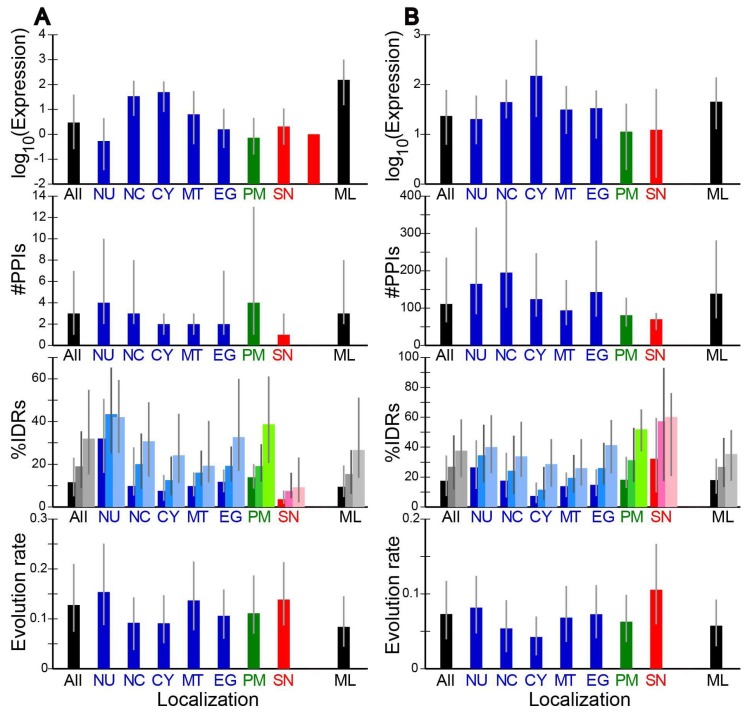
Medians of four quantities in different localizations in two non-mammalian eukaryotes. (**A**) *A. thaliana*; (**B**) *S. cerevisiae*; the data are presented as in Figure 1.

For each pair of orthologs, we determined the evolution rate, ω, defined by the ratio of nonsynonymous to synonymous substitution rate, i.e., dN/dS. The median ω at each localization is shown (Figure 1 and Figure 2). Note that for this and other data presented in the figure, different scales were used in different species. As the number of immune-related proteins (SI) in *A. thaliana* is small, no corresponding data were plotted in this species. Proteins of the four species showed similar patterns. For instance proteins in the cytosol (CY) and those that reside both in the nucleus and the cytosol (NC) had the median evolution rates lower than the overall median in all four species. In general the median evolution rates in intracellular proteins (NU, NC, CY, MT, and EG; shown in blue) were lower than those of secreted proteins (SN and SI; shown in red). Among the secreted proteins, immune-related proteins (SI) exhibited particularly high evolution rates, in agreement with the literature [13,14].

The fractional IDR content of each protein was predicted by DISOPRED [29], DICHOT [30], and POODLE-L [31] and the median in each localization was calculated (Figure 1 and Figure 2). Although the medians of most localizations (Figure 1 and Figure 2) were nearly always the lowest by DISOPRED, higher by DICHOT, and the highest by POODLE, we note that the overall averages by the three methods generally do not differ much. For instance, the fractions of IDRs in human proteins by DISOPRED, DICHOT, POODLE are 30.2%, 26.4%, and 30.1%, respectively. The differences in the medians are thus mostly attributable to differences in the distributions of fractional IDRs. Nevertheless the corresponding medians by the three prediction methods showed similar patterns. For instance, by all three methods in the four species, we got high fractional IDR contents in the nuclear proteins (NU) and low values in the mitochondrial proteins (MT), consistent with previous reports [7,8,9,10]. Intriguingly, the secreted non-immune proteins (SN) in budding yeast were revealed to have a high median IDR content, unlike the counterparts of the three multicellular eukaryotes. The difference may reflect the difference between unicellular and multicellular organisms. This issue needs to be addressed later with analyses of more eukaryotes.

We also calculated and graphed the median numbers of PPIs of proteins in the localizations (Figure 1 and Figure 2). PPIs have been less studied in mouse and thale cress proteins than in human and budding yeast counterparts, as evidenced by the reduced numbers of PPIs in mouse and thale cress (Figure 1B and Figure 2A). The mouse and thale cress PPI data are therefore less reliable as those of the other two species. As reported [28], multiply localized proteins (NC and ML) generally showed more interactions with other proteins. Immune-related secreted proteins (SI), however, had fewer interacting partners on average.

Additionally, the median expression level in ppm of the proteins at each localization was determined and graphed as logarithms to the base of ten (Figure 1 and Figure 2). Yeast proteins were generally expressed much more than mammalian proteins. The expression levels of the human immune-related proteins (SI) were generally high, but those of the mouse counterparts were indistinguishable from the average.

### 2.3. Correlation of Evolution Rates with Protein Properties

We computed Spearman’s correlation coefficients (Rhos) of number of PPIs with evolution rate (ω) and found them to be weakly negative but significantly different from zero (all at *p* < 0.01) (Table 2). The negative correlation is consistent with previous results [3,4]. As the number of PPIs was generally low in extracellular proteins (SN and SI, Figure 1 and Figure 2), the negative correlation partially explains their high evolution rates.

We also found small but significant (all at *p* < 1 × 10^−113^) negative correlations between expression level and **ω** (Table 2), corroborating previous findings [5,6]. The negative correlation was stronger in budding yeast. Since the expression levels of non-immune-related secreted proteins (SN) were not high (Figure 1 and Figure 2), the negative correlation at least in part explains the high evolution rates of these proteins. By contrast the expression levels of immune-related secreted proteins (SI) were not significantly low (Figure 1) and do not contribute to the extremely high evolution rates.

As IDRs have a propensity to evolve faster than SDs, the more IDRs a protein has, the faster it is expected to evolve. To test this possibility, correlation coefficients of %IDR with **ω** were calculated. Fractional IDR content was positively correlated with evolution rate in all the four species (Table 2). Although the correlation coefficients were generally small, they all significantly differed from zero (at *p* < 1 × 10^−4^). As the median fractional IDR contents in immune-related secreted proteins (SI) were lower than average, this factor does not make positive contribution to the evolution rates.

### 2.4. Evolution Rates in SDs and IDRs in Different Subcellular Localizations

**Figure 3:**
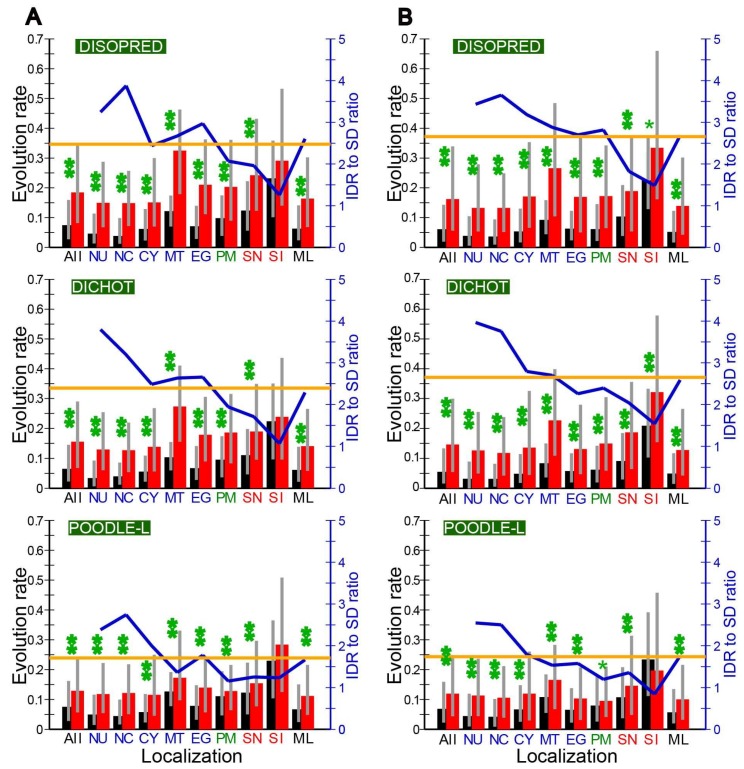
Evolution rates are higher in IDRs than in SDs except possibly for immune-related secreted proteins in mammals. (**A**) *H. sapiens*; (**B**) *M. musculus*; the diagrams (top to bottom) in each panel are based on DISOPRED, DICHOT and POODLE-L predictions. The median evolution rates in SDs are shown in black rectangles, while those in IDRs are depicted in red (left scale). Grey vertical lines show ranges from the 25th to the 75th percentile. Blue lines represent the median evolution rate ratios of IDRs to SDs at respective localizations, while horizontal orange lines show the ratio of all proteins (right scale). One asterisk signifies a statistically significant difference between the evolution rate distributions of IDRs and SDs at *p* < 0.01, while two asterisks denote a statistically significant difference at *p* < 0.001 (*U*-test). The same abbreviations for localizations as those in Table 1 are used.

In order to see whether IDRs or SDs in immune-related proteins mostly account for the high evolution rates, we calculated the evolution rates in IDRs and SDs separately and compared the two. The median evolution rate in IDRs in all proteins was significantly higher than that in SDs, irrespective of species (Figure 3 and Figure S1). We detected the same disparity at most localizations.

Upon closer examination of the mammalian rates, we noticed that the IDR/SD evolution rate ratio tended to be higher in intracellular localizations (NU, NC, CY, MT, and EG) than in extracellular ones (SN and SI). In the plant *A. thaliana* the inside–outside difference in evolution rate was detectable but was less pronounced (Figure S1A). In contrast, budding yeast failed to show this tendency (Figure S1B). In immune-relate secreted proteins (SI), the rates in IDRs and SDs were both higher than average, with the difference between them statistically insignificant in a majority of cases (Figure 3). SDs apparently evolve quite rapidly in immune-related proteins to approach the rates of IDRs to give rise to the anomalously high evolution rates. So far as we are aware, the phenomenon of the evolution rate in SDs that comes close that in IDRs in immune-related proteins is the first to be reported. The non-immune related extracellular proteins (SN) also tended to have higher than average evolution rate in SDs in *H. sapiens* and *M. musculus*, and *A. thaliana*, although the difference from the average was more conspicuous in the two mammals (Figure 3) than in the plant species (Figure S1A). In contrast SDs in non-immune related extracellular proteins (SN) did not show an above-average evolution rate in *S. cerevisiaie* (Figure S1B). In mammalian mitochondrial (MT) and plasma membrane (PM) proteins, the evolution rates of SDs and IDRs were close to each other (Figure 3), although the former was significantly higher than the latter in all cases. By contrast the counterparts in the two non-mammalian species failed to show the tendency (Figure S1). We need to investigate other species before attaching any significance to this possibly mammalian-specific phenomenon.

We recognize the need to analyze more animal species to check the generality of our finding on immune-related extracellular proteins. For accurate analyses by the same methodology, however, two closely related and entirely sequenced species must be available and at least one of them must have a majority of proteins annotated by UniProt to provide reliable subcellular localizations. Unfortunately no animal species other than *H. sapiens* and *M. musculus* currently meet the latter criterion. Since 3463 (~22% of the total) *Drosophila melanogaster* proteins have been annotated, however, we carried out preliminary analyses of this fly. Thirty-eight annotated immune-related extracellular proteins were identified in 13,957 orthologs. The results showed that the evolution rates in IDRs and SDs were both high in immune-related proteins but the former was much higher than the latter. The ratio of the median evolution rate in IDRs to that in SDs was 2.37, 1.60, and 2.99 by DISOPRED, DICHOT, and POODLE, respectively. As the corresponding ratios of all *Drosophila* proteins were 2.10, 2.45, and 1.79, the ratio was not necessarily diminished in immune-related proteins in fruit fly. Thus, the preliminary results indicate that the phenomenon of rapid evolution in both SD and IDRs in immune-related secreted proteins is possibly limited to vertebrates.

In the cytosolic proteins (CY) of budding yeast, the median evolution rate in IDRs was only a little higher than that in SDs (Figure S1B). As noted before, budding yeast proteins generally interact with much more proteins than human proteins and did not exhibit intracellular-extracellular disparity in the IDR to SD evolution ratio.

### 2.5. Examles of Proteins with Nonsynonymous and Synonymous Substitutions

**Figure 4:**
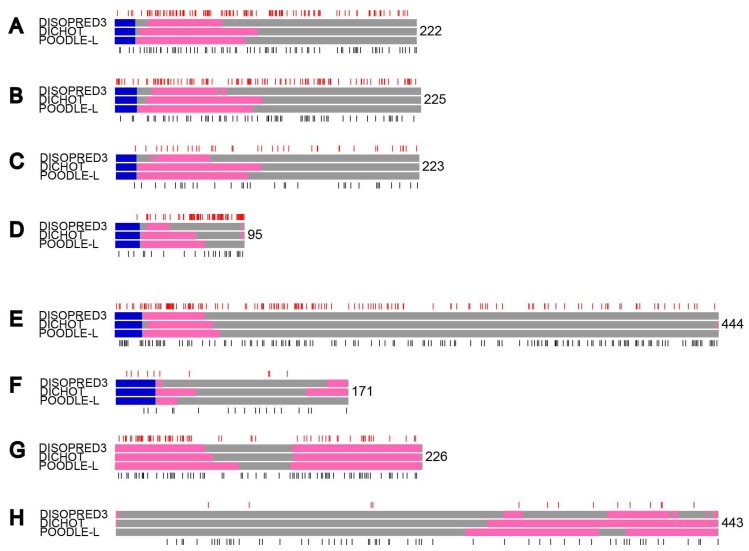
Examples of proteins with locations of nonsynonymous and synonymous substitutions. Each protein is represented by three rectangles with DISOPRED, DICHOT, and POODLE predictions (top to bottom) of IDRs (pink) and SDs (gray) as well as signal sequences (blue), if any, and the length shown on the right. The locations of nonsynonymous and synonymous substitutions are shown above (red lines) and below (black lines), respectively. (**A**–**D**): Immune-related secreted proteins, (**E**,**F**): non-immune-related secreted proteins, (**G**,**H**): nuclear proteins. (**A**) The human PRG2, (**B**) human PRG3, (**C**) mouse PRG2, (**D**) mouse DEFA20, (**E**) human SERPINA10, (**F**) mouse NENF, (**G**) human PROP1, (**H**) mouse NEK2 proteins.

To give specific examples, we diagramed some human and mouse proteins with locations of nonsynonymous and synonymous substitutions (Figure 4). As we selected the proteins as they exhibit close-to-median ratios of nonsynonymous to synonymous substitution rates in SDs and IDRs, the frequencies of nonsynonymous to synonymous substitutions do not necessarily show median values. Although the three prediction methods gave different results, the major disparities were found in the boundaries of IDRs and did not affect main results. In immune-related secreted proteins (Figure 4A–D), nonsynonymous mutations (red bars) were almost as frequent as synonymous ones (black bars) both in IDRs (pink rectangles) and SDs (gray regions). In comparison, in proteins of other subcellular localizations, nonsynonymous substitutions occurred much less frequently than synonymous substitutions in SDs, while the difference was less pronounced in IDRs (Figure 4E–H).

### 2.6. Significance and Remaining Issues

The generally much lower frequency of nonsynonymous substitutions than synonymous substitutions in SDs reflects the fact that nonsynonymous changes very often destabilize the structures. By contrast, the difference between nonsynonymous and synonymous substitution rates is diminished in IDRs as nonsynonymous changes are frequently accommodated in IDRs. Consequently the ratio of nonsynonymous to synonymous substitution rate (ω) is expected to be much smaller in SDs than in IDRs. Although the actual results obtained in this research were mostly consistent with this expectation, those of immune-related secreted proteins of the two mammalian species were not; ω in SDs approaches that in IDRs.

As ω is larger than 1 at positively selected sites, the existence of numerous such sites in a region increases the average ω. Since many sites in immune-related secreted proteins were reported to be under positive selection [13,14,15,16,17], the heightened ω in SDs of such proteins indicates that many positively selected sites fall in SDs. The observation that IDRs of immune-related proteins exhibit higher ω than those of other proteins also implies that IDRs contain positively selected sites, too. The classification of positively selected sites in immune-related proteins into SDs and IDRs will probably lead to a better understanding of mechanisms of immunity. It is plausible that many nonsynonymous changes occur at the surface of SDs that interacts with other proteins.

It is also of interest to investigate known genes under positive selection that are associated with gamete recognition [32,33] and male reproduction [34,35] to find if SDs as well as IDRs of the encoded proteins evolve rapidly. We note that extracellular domains receive a number of posttranslational modifications such as phosphorylations, glycosylation, and lipidation. Investigation of evolution rates at posttranslational modification sites of immune-related proteins is another prospective area.

## 3. Materials and Methods

The nucleotide sequences of *H. sapience*, *M. musculus,* and *Rattus norvegicus* genes were downloaded from Ensembl (Release 91) [36]. The nucleotide sequences of *A. thaliana* (TAIR10), *Arabidopsis lyrata*, *Drosophila melanogaster* (BDGP6) genes were obtained from Ensembl, too. Ensembl also provided the orthologous relationships between *H. sapience* and *M. musculus* as well as those of *M. musculus* and *R. norvegicus*. The sequences of *S. cerevisiae* and *Saccharomyces paradoxus* were obtained from the Saccharomyces Genome Database [37], while those of *Drosophila pseudoobscura* genes were downloaded from FlyBase [38]. The orthologs of the two *Arabisopsis* species, the two yeast species, and the two *Drosophila* species were selected by bidirectional best hit analysis. The proteins were classified by subcellular localizations based on the Gene-Ontology (GO) annotations in UniProt (Release 2017_05) [39]. Specifically, the following GO IDs were used for subcellular classifications: nucleus: GO:0005634; cytoplasm: GO:0005829; mitochondria: GO:0005739; endoplasmic reticulum/Golgi apparatus: GO:0005783, GO:0005794, and GO:0005793; plasma membrane: GO:0005886; secreted: GO:0005576 and GO:0005615; immune-related: GO:0002376.

From the coding sequences, the signal peptides were removed based on UniProt annotations because they are unclassifiable as SDs or IDRs due to their absence in mature proteins. The remaining amino acid sequences of orthologs were aligned by MAFFT [40] and the corresponding nucleotide sequences were aligned according to the MAFFT results. dn/ds values were then computed using the codeml program (model M0) in PAML (version 4.9d) [41]. Statistical differences between two quantities were tested by Mann-Whitney’s *U*-test, while statistical significance of correlations was evaluated by Spearman’s rank correlation by means of in-house programs.

Number of PPIs and expression levels were taken from the BioGRID (version 3.4.158) [42] and the PaxDb (version 4.1) [43] databases, respectively. BioGRID is a curated database of interactions including protein-protein interactions obtained by two-hybrid studies, affinity purification coupled to mass spectrometry, and other methods, while PaxDB contains whole genome protein abundance information obtained by integrating numerous datasets using scores and ranks. Each protein was divided into SDs and IDRs by three methods: DISOPRED3 [29], DICHOT [30] and POODLE-L [31]. Briefly, DISOPRED3 assigns IDRs based on sequence profiles and other sequence-derived features, DICHOT classifies proteins into SDs and IDRs using sequence characteristics, alignments to existing protein structures, and sequence divergence, while POODLE-L is a prediction method for long IDRs that makes use of support-vector machine with 10 kinds of simple physico-chemical properties of amino acids. Based on the overall MAFFT alignments, the alignments of the corresponding sections were made. The evolution rate of each section was then determined as above.

## 4. Conclusions

In human and mouse, the SDs of immune-related proteins evolve at a high rate which comes close to that of the IDRs. This observation indicates that positive selection operates on both SDs and IDRs of the encoded proteins in many immune-related genes. Comparatively high evolution in SDs is also observed in non-immune-related secreted proteins in human and mouse, and to a lesser extent in thale cress, but not in budding yeast. Thus accelerated evolution in SDs as well as in IDRs contributes to rapid evolution of extracellular proteins in mammals.

## Figures and Tables

**Table 1:** Number of proteins in each subcellular localization.

Species	All	NU	NC	CY	MT	EG	PM	SN	SI	ML
*H. sapiens*	10,348	1639	632	455	377	400	1116	584	139	3023
*M. musculus*	10,068	1719	546	224	426	514	998	796	125	2787
*A. thaliana*	8910	1032	163	331	356	348	534	431	6	594
*S. cerevisiae*	5304	1532	232	241	639	458	281	69	0	416

NU: Nucleus; NC: Nucleus and cytosol; CY: Cytosol; MT: Mitochondria; EG: Endoplasmic reticulum or Golgi apparatus; PM: Plasma membrane; SN: Secreted, non-immune-related; SI: Secreted, immune-related; ML: Multiple localizations except NC (ML).

**Table 2:** Correlations between three properties and evolution rate **ω**.

Correlation with	*H. sapiens*	*M. musculus*	*A. thaliana*	*S. cerevisiae*
#PPI with ω	−0.293	−0.194	−0.054	−0.195
Expression level with ω	−0.264	−0.231	−0.337	−0.459
%IDR (DISOPRED) with ω	0.093	0.094	0.168	0.264
%IDR (DICHOT) with ω	0.113	0.146	0.052	0.303
%IDR (POODLE) with ω	0.096	0.097	0.113	0.179

Spearman’s correlation coefficient (Rho) of each pair is shown.

## Supplementary Materials

The following are available online at http://www.mdpi.com/1422-0067/19/12/3860/s1.

## Abbreviations

SD	Structural domain
IDR	Intrinsically disordered region
PPI	Protein-to-protein interaction
NU	Nucleus
NC	Nucleus and cytosol
CY	Cytosol
MT	Mitochondria
EG	Endoplasmic reticulum or Golgi apparatus
PM	Plasma membrane
SN	Secreted, non-immune-related
SI	Secreted, immune-related
ML	Multiple localizations except NC
dN	Nonsynonymous substitution rate
dS	Synonymous substitution rate
GO	Gene Ontology
