# Supplementary material for: Both Intrinsically Disordered Regions and Structural Domains Evolve Rapidly in Immune-Related Mammalian Proteins
Source: Int J Mol Sci. 2018 Dec 4;19(12):3860. doi: 10.3390/ijms19123860 (PMC6321239; doi:10.3390/ijms19123860)
Supplement: Supplementary file 1 [file ijms-19-03860-s001.pdf]

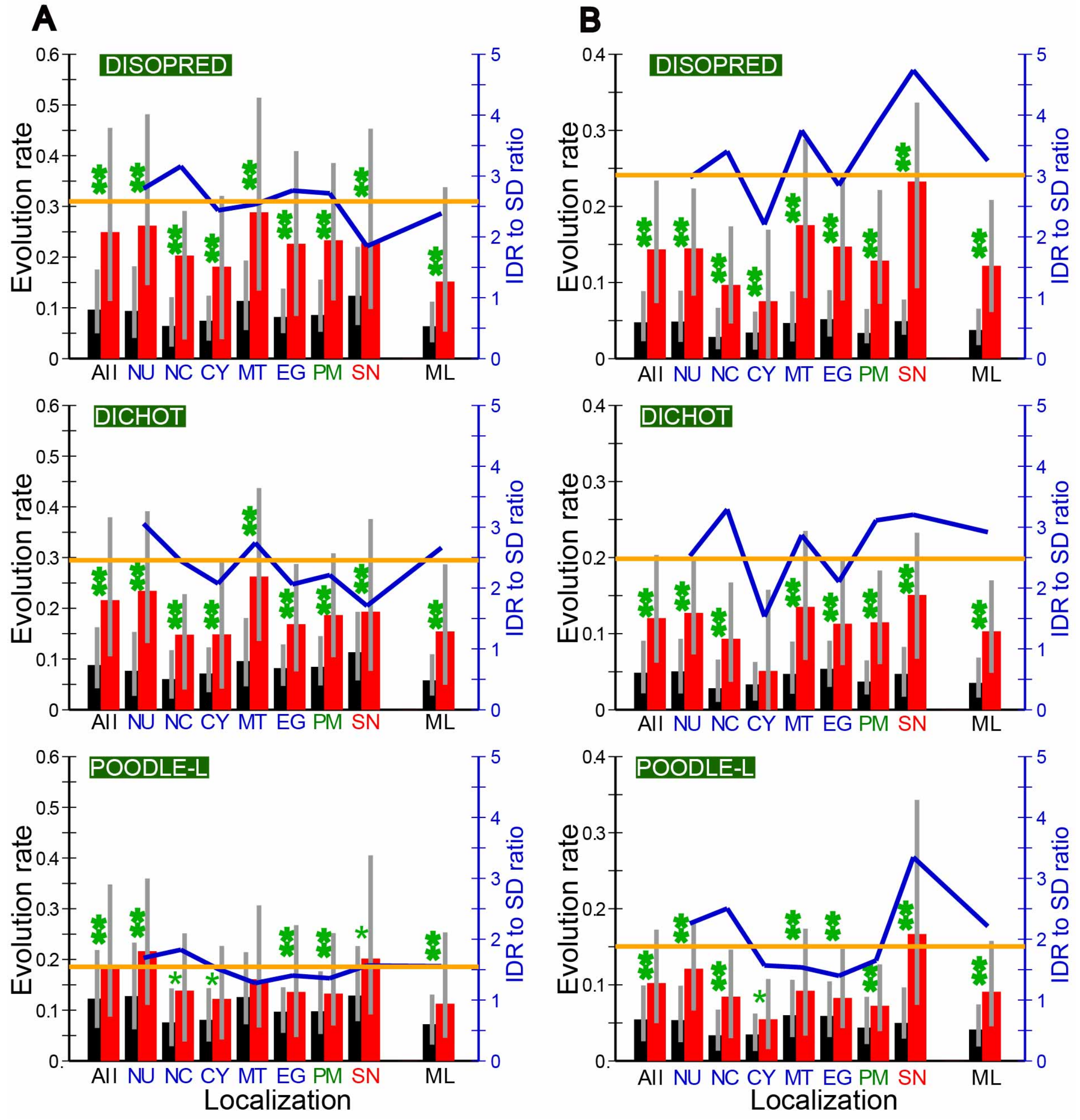

Figure S1. Evolution rates are higher in IDRs than in SDs in non-mammalian eukaryotes. (**A**) *A. thaliana*; (**B**) *S. cerevisiae*; the data are presented as in Fig. 3, except that no data of non-immune related extracellular proteins (SI) are shown.
